# Quantum Renormalization of Spin Squeezing in Spin Chains

**DOI:** 10.1038/s41598-018-35666-z

**Published:** 2018-12-12

**Authors:** Leila Balazadeh, Ghader Najarbashi, Ali Tavana

**Affiliations:** University of Mohaghegh Ardabili, Physics, ardabil, P.O. Box 179 Iran

## Abstract

By employing quantum renormalization group (QRG) method, we investigate quantum phase transitions (QPT) in the Ising transverse field (ITF) model and in the XXZ Heisenberg model, with and without Dzyaloshinskii Moriya (DM) interaction, on a periodic chain of N lattice sites. We adopt a new approach called spin squeezing as an indicator of QPT. Spin squeezing, through analytical expression of a spin squeezing parameter, is calculated after each step of QRG. As the scale of the system becomes larger, (after many QRG steps), the ground state (GS) spin squeezing parameters show an abrupt change at a quantum critical point (QCP). Moreover, in all of the studied models, the first derivative of the spin squeezing parameter with respect to the control parameter is discontinuous, which is a signature of QPT. The spin squeezing parameters develop their saturated values after enough QRG iterations. The divergence exponent of the first derivative of the spin squeezing parameter in the near vicinity of the QCP is associated with the critical exponent of the correlation length.

## Introduction

Quantum phase transition (QPT), has been of great interest to the condensed matter physicists in recent decades. It is a continuous phase transition at or near absolute zero temperature driven by changing an internal or an external parameter of the Hamiltonian of the system^1–7^. QPT entails a drastic change in the GS properties of a system at the QCP. Precise identification of QCPs is of fundamental importance, studying the critical behaviour of the strongly correlated systems in condensed matter physics. Quantum fluctuations at zero temperature, in the absence of thermal fluctuations, lead to QPT. This is the motivation to the basic idea of the quantum information theory approach to the detection of QPTs.

In the past few years, efforts had been made to investigate the QPT in different spin models using quantum correlation functions. In particular, study of the quantum entanglement as an effective indicator of QPT, has received considerable attention^8–14^. In addition, it has been proven that other quantum correlations such as quantum discord^15,16^, fidelity^17^, entanglement entropy^18^, monogamy property^19,20^, etc., can also be used to describe the QPT in the quantum critical systems.

Recently, it has been proposed to implement the QRG method, introduced by Wilson in 1975^21^, to investigate the nonanalytic behaviour of quantum correlations of many-body systems, such as entanglement measures, quantum discord, fidelity and monogamy property in the vicinity of QCPs^22–33^. By establishing a projection of low-lying eigenstates of a *l*-size block of a *N*-size system, the whole system reduces to a *N*/*l*-size space. The block decimation of the Kadanoff’s block method is a common feature of different QRG methods^34–36^. The density matrix RG (DMRG) method uses a numerical algorithm to find the ground state in one dimensional quantum systems, e.g. the Heisenberg or Bose-Hubbard models^37^. While attempts have been done to apply the DMRG to two-dimensional and some small three-dimensional clusters, it fails for the larger systems and higher dimensions. DMRG, investigates the flows in the density matrix space, unlike other QRG method that deal with the Hamiltonian of the system to find the critical behaviors of the whole system by decimation. Even though the QRG method reduces the degrees of freedom of the system, but it gives acceptable results comparable with the results of analytical calculations, DMRG, multi-scale entanglement renormalization ansatz (MERA)^38^ and projected entangled pair states (PEPS)^39^. Each of the mentioned approaches has its own advantages and limitations. For example, despite of the successful applications of QRG and its simplicity, it fails in the description of strongly correlated systems and some spin models such as the one-dimensional bilinear biquadratic spin-1 model. Independence of the correlation length from the system in the near vicinity of the QCPs, makes the QRG efficient in studying the model systems. The simplicity of the QRG is a vital advantage in studying complicated models with several interactions in higher spins, higher dimensions and in complex geometries. Studying the renormalization of quantum correlations may help to clarify the QPTs in many-body systems. Therefore, it is helpful to investigate the renormalization of various quantum correlation measures as indicators of QPT in every system.

Spin squeezing, connected to the quantum correlations between the spins, is one of the most successful approaches to detect the multipartite entanglement in many-spin systems^40–45^. Because of important applications, spin squeezing has attracted significant attention as a subject of theoretical and experimental investigations^46–48^. The notion of spin squeezing has many advantages where the most important one is the simplicity in generating and measuring it, for instance, in atomic interferometry, weak magnetic fields^49,50^, spin noise in quantum fluids^51,52^, magnetometry with a spinor Bose-Einstein condensate^53^, Ramsey spectroscopy, atom clocks and in quantum computing^54–59^. Specially, it improves the precision of experimental measurements^60–62^. Another advantage is that the spin squeezing is a multipartite entanglement witness^14,42,63,64^. Moreover, the sensitivity of a state with respect to SU(2) rotations can be characterized by the spin squeezing parameters^65^. Measuring spin squeezing in a many-spin system needs no bipartition or reduction process, unlike some other measures of entanglement, e.g. concurrence or negativity. So, it is easy to be used for every spin model, independent of size, dimension and geometry of the system. Among various definitions of the spin squeezing parameter^40,42^, here we use the most widely studied one, defined by Kitagawa and Ueda^40^.

To the best of our knowledge, there is no report on the renormalization of a spin squeezing parameter in detection of QPT in spin chains, till now. Therefore, our main purpose in this work is to use the QRG method to study the scaling behaviour of the spin squeezing parameter in the vicinity of QCP in some spin-1/2 models.

The paper is organized as follows; We apply the QRG method on a spin-1/2 ITF chain model to investigate the scaling behaviour of the spin squeezing parameter. Then, we briefly review the renormalization of the one dimensional anisotropic XXZ model with DM interaction. We aim to investigate the behaviour of the spin squeezing parameter in detecting QPTs. We also study the renormalization of the spin squeezing parameter in the one-dimensional anisotropic XXZ-Heisenberg model.

## Renormalization of the ITF Model

**Figure 1:**
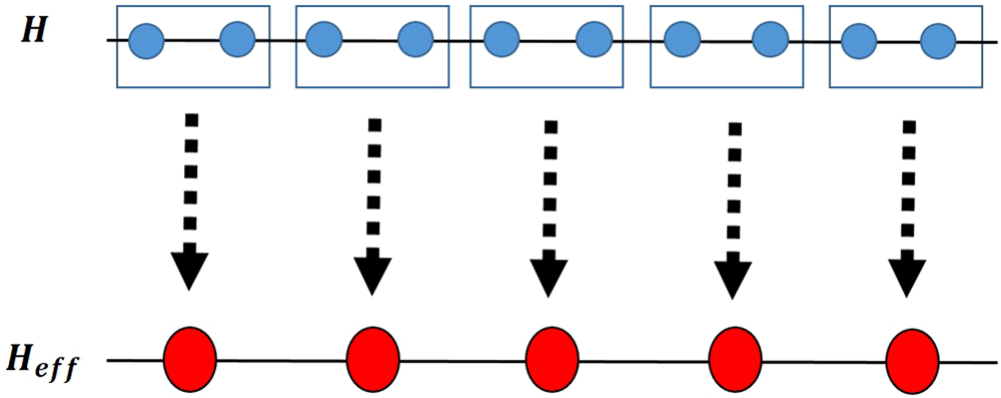
A representation of the Kadanoff’s block QRG method by recomposing a one-dimensional spin chain into a chain of two-spin blocks.

The Hamiltonian of the ITF model^66,67^ on a periodic chain of N spin-1/2 sites is

1 $$H=-\,J\,\sum _{i=1}^{N}\,({\sigma }_{i}^{x}{\sigma }_{i+1}^{x}+\lambda {\sigma }_{i}^{z}),$$

where *i* is the number of the site, *J* denotes the nearest-neighbor coupling that scales the energy, *λ* is the strength of transverse magnetic field and ${\sigma }_{i}^{\alpha }$ (*α* = *x*, *y*, *z*) are the usual Pauli matrices of the *i*-th spin. From the exact solution^68,69^ it can be seen that the value of the order parameter, i.e. the magnetization, in the ground state of this model changes from the non-null value for *λ* < 1, i.e. the ferromagnetic phase, to the null value for *λ* > 1, in the paramagnetic phase. In other words, system exhibits QPT with the QCP *λ*_*c*_ = 1. In order to employ the idea of QRG approach, we use the Kadanoff’s block method for one dimensional spin systems and divide the N-spin chain into N/2 two-spin blocks as shown in Fig. 1.

Then, the total Hamiltonian is rewritten in two parts as

2 $$H={H}^{B}+{H}^{BB},$$

where the intrablock Hamiltonian, ${H}^{B}=\sum _{I=1}^{N/2}\,{h}_{I}^{B}$, is the summation of the block Hamiltonian, ${h}_{I}^{B}$, where

3 $${h}_{I}^{B}=-\,J({\sigma }_{1,I}^{x}{\sigma }_{2,I}^{x}+\lambda {\sigma }_{1,I}^{z}).$$

The second part is the interblock interactions and is equal to

4 $${H}^{BB}=-\,J\,\sum _{I=1}^{N/2}\,({\sigma }_{2,I}^{x}{\sigma }_{1,I+1}^{x}+\lambda {\sigma }_{2,I}^{z}).$$

Here, ${\sigma }_{\mathrm{1,}I}^{\alpha }$ denotes the *α*-the component of the Pauli matrices at the first site in the *I*-th block. The matrix form of the two-spin block Hamiltonian is

5 $${h}_{I}^{B}=(\begin{array}{cccc}-J\lambda  & 0 & 0 & -J\\ 0 & -J\lambda  & -J & 0\\ 0 & -J & J\lambda  & 0\\ -J & 0 & 0 & J\lambda \end{array}),$$

the eigenstates and corresponding eigenvalues are:

6 $$\begin{array}{ll}|{e}_{1}\rangle =\frac{1}{\sqrt{1+{q}^{2}}}(q|\uparrow \uparrow \rangle +|\downarrow \downarrow \rangle ), & {E}_{1}=-\,J\sqrt{1+{\lambda }^{2}},\\ |{e}_{2}\rangle =\frac{1}{\sqrt{1+{q}^{2}}}(q|\uparrow \downarrow \rangle +|\downarrow \uparrow \rangle ), & {E}_{2}=-\,J\sqrt{1+{\lambda }^{2}},\\ |{e}_{3}\rangle =\frac{1}{\sqrt{1+{r}^{2}}}(r|\uparrow \uparrow \rangle +|\downarrow \downarrow \rangle ), & {E}_{3}=J\sqrt{1+{\lambda }^{2}},\\ |{e}_{4}\rangle =\frac{1}{\sqrt{1+{r}^{2}}}(r|\uparrow \downarrow \rangle +|\downarrow \uparrow \rangle ), & {E}_{4}=J\sqrt{1+{\lambda }^{2}},\end{array},$$

where $q=\lambda +\sqrt{1+{\lambda }^{2}}$, $r=\lambda -\sqrt{1+{\lambda }^{2}}$, and $|\,\uparrow \,\rangle$ and $|\,\downarrow \,\rangle$ are the eigenstates of the Pauli matrix *σ*^*z*^.

Then, we can construct the projection operator using two eigenstates of ${h}_{I}^{B}$ with lowest eigenvalues, as ${P}_{0}^{I}=|{e}_{1}{\rangle }_{I}{\langle \Uparrow |+|{e}_{2}\rangle }_{I}\langle \Downarrow |$, where $\langle \Uparrow |$ and $\langle \Downarrow |$ are the renormalized states of the *I*-th block.

The projection operator of the whole system can be defined as

7 $${P}_{0}=\prod _{I=1}^{N/2}\,{P}_{0}^{I}.$$

The effective Hamiltonian can be constructed by applying the projection operator to the original Hamiltonian

8 $${H}_{eff}={P}_{0}H{P}_{0}.$$

Renormalization of the Pauli matrices at the first and the second sites are given by

9 $$\begin{array}{rcl}{P}_{0}{\sigma }_{1,I}^{x}{P}_{0} & = & 2ab{\tilde{\sigma }}_{I}^{x},\\ {P}_{0}{\sigma }_{2,I}^{x}{P}_{0} & = & ({a}^{2}+{b}^{2}){\tilde{\sigma }}_{I}^{x},\\ {P}_{0}{\sigma }_{1,I}^{z}{P}_{0} & = & ({a}^{2}-{b}^{2})I,\\ {P}_{0}{\sigma }_{2,I}^{z}{P}_{0} & = & ({a}^{2}-{b}^{2}){\tilde{\sigma }}_{I}^{z},\end{array}$$

with $a=\frac{q}{\sqrt{1+{q}^{2}}}$ and $b=\frac{1}{\sqrt{1+{q}^{2}}}$. ${\tilde{\sigma }}_{I}^{x}$, ${\tilde{\sigma }}_{I}^{z}$ and *I* are the new renormalized block operators in the renormalized Hilbert space that are defined as ${\tilde{\sigma }}_{I}^{z}=|\Uparrow {\rangle }_{I}{\langle \Uparrow |-|\Downarrow \rangle }_{I}\langle \Downarrow |$, ${\tilde{\sigma }}_{I}^{x}=|\Uparrow {\rangle }_{I}{\langle \Downarrow |+|\Downarrow \rangle }_{I}\langle \Uparrow |$ and $I=|\Uparrow {\rangle }_{I}{\langle \Uparrow |+|\Downarrow \rangle }_{I}\langle \Downarrow |$.

We can collect all these relations together and obtain the full renormalized Hamiltonian after one step of renormalization,

10 $${H}^{(1)}=-\,{J}^{(1)}\,\sum _{i=1}^{N/2}\,({\tilde{\sigma }}_{i}^{x}{\tilde{\sigma }}_{i+1}^{x}+{\lambda }^{(1)}{\tilde{\sigma }}_{i}^{z}).$$

It is clear that, our choice of QRG transformation preserves the form of the original Hamiltonian, so *J*^(1)^ and *λ*^(1)^ are the new renormalized coupling constant and the strength of the transverse magnetic field. One obtains the following iterative relations

11 $${J}^{(1)}=\frac{J}{\sqrt{1+{\lambda }^{2}}},\,{\lambda }^{(1)}={\lambda }^{2},$$

After setting *λ*^(1)^ = *λ*, the resulting fixed point equation is λ* = λ*^2^ with a nontrivial fixed point which is the critical point of the ITF model, i.e. *λ*_*c*_ = 1. These results were deduced before in several studyings^2,23,30,31^. The *n*-fold renormalization of the coupling constants can be obtained for a chain with $N={n}_{B}^{n+1}$ spins, where *n*_*B*_ is the number of spins in a block, in the Kadanoff’s block method. For the ITF model, *n*_*B*_ = 2.

12 $${\lambda }^{(n+1)}={({\lambda }^{(n)})}^{2},\,{J}^{(n+1)}=\frac{{J}^{(n)}}{\sqrt{1+{({\lambda }^{(n)})}^{2}}},$$

## Renormalization of the Spin Squeezing Parameter in the ITF Model

In this section, we study the spin-1/2 squeezing parameter in the GS of the ITF model. First, we briefly review the definition of the spin squeezing parameter ${\xi }_{S}^{2}$ according to ref. ^40^,

13 $${\xi }_{S}^{2}=\frac{4{({\rm{\Delta }}{\overrightarrow{J}}_{{\mathop{n}\limits^{\rightharpoonup }}_{\perp }})}_{{\rm{\min }}}^{2}}{N},$$

where *N* is the number of spins on the chain and ${\mathop{n}\limits^{\rightharpoonup }}_{\perp }$ refers to the direction perpendicular to the mean spin direction. Minimization will be done over all directions. ${\overrightarrow{J}}_{{\mathop{n}\limits^{\rightharpoonup }}_{\perp }}$ is $\overrightarrow{J}$ along the ${\mathop{n}\limits^{\rightharpoonup }}_{\perp }$ and $\overrightarrow{J}=({J}^{x},{J}^{y},{J}^{z})$ denotes the angular momentum operator of an ensemble of spin-1/2 particles. If ${\xi }_{S}^{2} < 1$, the state is squeezed while for the coherent spin state (CSS), ${\xi }_{S}^{2}$ is equal to 1. A spin squeezed state, i.e. ${\xi }_{S}^{2} < 1$, is pairwise entangled, while a pairwise entangled state may not be a spin-squeezed state, according to the squeezing parameter ${\xi }_{S}^{2}$^42^. The components of $\overrightarrow{J}$ for a *N* spin-1/2 chain is given by

14 $${J}^{\alpha }=\frac{\hslash }{2}\,\sum _{i=1}^{N}\,{\sigma }_{i}^{\alpha },\,\alpha =\{x,y,z\},$$

We assume that $\hslash =1$ for the sake of simplicity.

In continue, we choose one of the degenerated GSs, i.e. |*e*_1_〉, and calculate the ${\xi }_{S}^{2}$ for this state by substituting *N* = 2 in the relations. The first step to calculate the parameter ${\xi }_{S}^{2}$ is to calculate the mean spin direction which is obtained as

$$(\langle {e}_{1}|{J}^{x}|{e}_{1}\rangle ,\langle {e}_{1}|{J}^{y}|{e}_{1}\rangle ,\langle {e}_{1}|{J}^{z}|{e}_{1}\rangle )=(0,0,1).$$

So, we can write ${\mathop{n}\limits^{\rightharpoonup }}_{\perp }=\,\cos \,(\phi )(1,0,0)+\,\sin \,(\phi )(0,1,0)$ where, *φ* is an arbitrary angle and the following variance is minimized over *φ*.

15 $${({\rm{\Delta }}{\overrightarrow{J}}_{{\mathop{n}\limits^{\rightharpoonup }}_{\perp }})}_{{\rm{\min }}}^{2}={(\langle {e}_{1}|{({\overrightarrow{J}}_{{\mathop{n}\limits^{\rightharpoonup }}_{\perp }})}^{2}|{e}_{1}\rangle -{(\langle {e}_{1}|{\overrightarrow{J}}_{{\mathop{n}\limits^{\rightharpoonup }}_{\perp }}|{e}_{1}\rangle )}^{2})}_{{\rm{\min }}}.$$

After calculations, we obtain

16 $${\xi }_{S}^{2}=1-\frac{1}{\sqrt{1+{\lambda }^{2}}},$$

it can be seen that ${\xi }_{S}^{2}$ is a function of the strength of the transverse magnetic field. Consequently, the spin squeezing parameter in the *n*-th step of the QRG can be written as

17 $${({\xi }_{S}^{2})}^{(n)}=1-\frac{1}{\sqrt{1+{({\lambda }^{(n)})}^{2}}}.$$

By substituting *λ*_*c*_ = 1 into the Eq. (12), we get ${({\xi }_{S}^{2})}_{{\lambda }_{c}}^{(n)}=0.3$. In other words, *λ*_*c*_ = 1 is the fixed point of the spin squeezing parameter of all different QRG steps.

**Figure 2:**
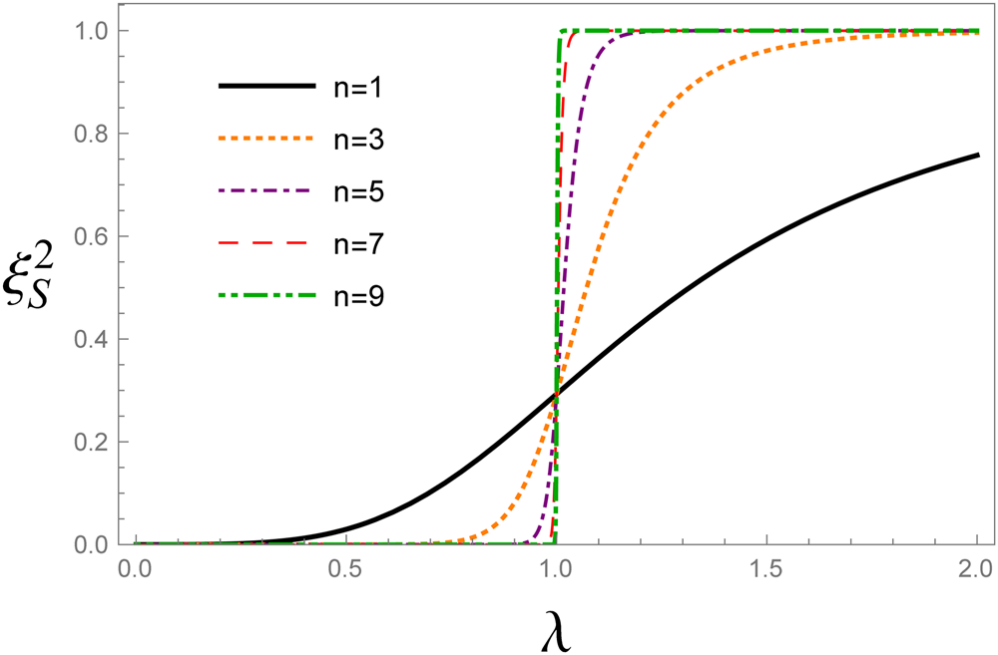
The evolution of the spin squeezing parameter ${\xi }_{S}^{2}$ under the QRG iterations *n*, versus the transverse magnetic field *λ* in the ITF model.

**Figure 3:**
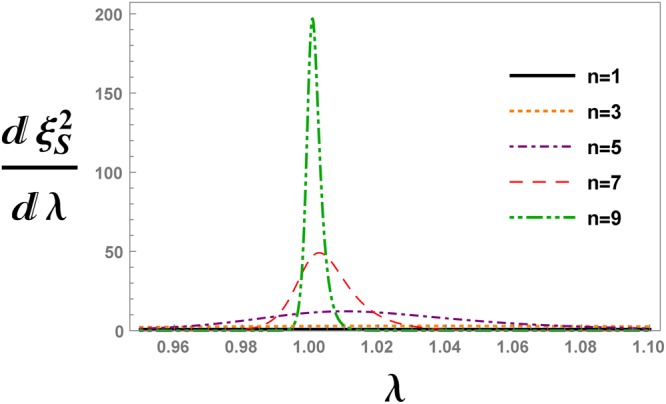
The evolution of the first derivative of the spin squeezing parameter ${\xi }_{S}^{2}$ with respect to the transverse magnetic field *λ*, after *n*-th QRG iteration in the ITF model.

**Figure 4:**
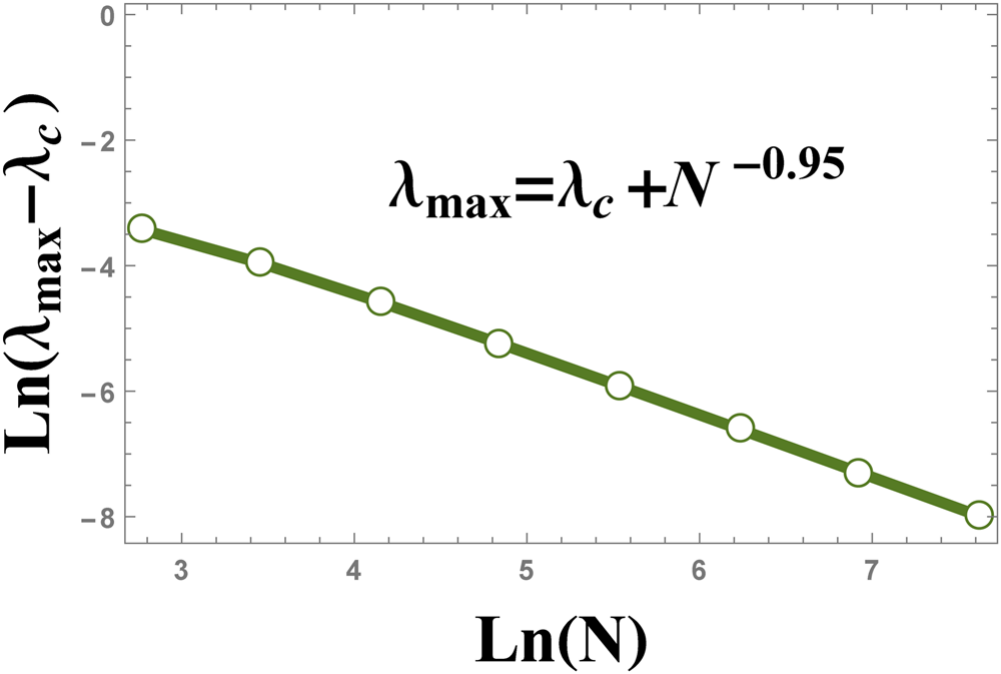
The scaling behaviour of $|{\lambda }_{{\rm{\max }}}-{\lambda }_{c}|$ versus the size of the system *N*, where *λ*_max_ is the position of the peak in Fig. 3.

**Figure 5:**
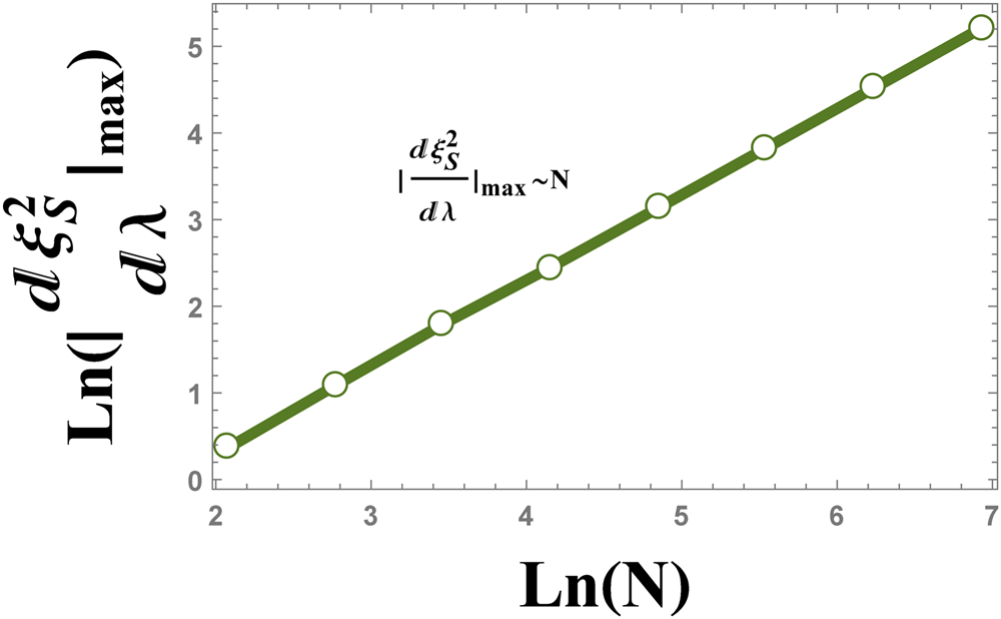
The logarithmic plot of the absolute value of ${\tfrac{d{\xi }_{S}^{2}}{d\lambda }|}_{{\rm{\max }}}$ versus the size of the system *N*. The plot is linear corresponding to the scaling behaviour ${|\tfrac{d{\xi }_{S}^{2}}{d\lambda }|}_{{\rm{\max }}}\,\sim \,{N}^{0.99}$.

Spin squeezing parameter versus transverse magnetic field is plotted in Fig. 2 for different QRG steps. The cross point appearing in Fig. 2, represents the QCP, *λ*_*c*_ = 1. Spin squeezing parameter develops two different saturated values; ${\xi }_{S}^{2}=0$ for *λ* < 1 and ${\xi }_{S}^{2}=1$ for *λ* > 1. In Fig. 2, the QCP is detected by iterative renormalization steps which justifies that the spin squeezing parameter can be used as an indicator of QPT. We have plotted the first derivative of the spin squeezing parameter with respect to the transverse magnetic field, i.e. $\frac{d{\xi }_{S}^{2}}{d\lambda }$, at different QRG steps, in Fig. 3. From the figure, it is obvious that there are peaks in the $\frac{d{\xi }_{S}^{2}}{d\lambda }$ plot at the points *λ*_max_, that tend to *λ*_*c*_ with increasing QRG steps, i.e. the thermodynamic limit. In Fig. 4, the position of $\frac{d{\xi }_{S}^{2}}{d\lambda }$ peak versus the size of the system is plotted. It can be seen from the scaling behaviour *λ*_max_ = *λ*_*c*_ + *N*^−0.95^, that *λ*_max_ goes to *λ*_*c*_ at the thermodynamic limit, *N* → ∞. So, *λ*_max_ scales as $|{\lambda }_{{\rm{\max }}}-{\lambda }_{c}|={N}^{-\theta }$, where *θ* = −0.95. The numerical results plotted in Fig. 5, show the behaviour of the absolute value of the maximum of $\frac{d{\xi }_{S}^{2}}{d\lambda }$, versus the size of the system, *N*, as ${|\frac{d{\xi }_{S}^{2}}{d\lambda }|}_{{\lambda }_{{\rm{\max }}}}\,\sim \,{N}^{\theta }$. The exponent of the scaling behaviour is *θ* = 0.99. It can be shown that the exponent *θ* is related to the critical exponent of the correlation length, as it is shown in the case of the concurrence measure by Kargarian *et al*.^23^. Close to the critical point, *λ*_*c*_, the correlation length diverges with the exponent *ν*, i.e., $\zeta \sim |\lambda -{\lambda }_{c}{|}^{-\nu }$. This behaviour is seen for every QRG step, ${\zeta }^{(n)}\sim |{\lambda }^{(n)}-{\lambda }_{c}{|}^{-\nu }$. From the Kadanoff’s block method, ${\zeta }^{(n)}=\frac{\zeta }{{2}^{n}}$ that leads to ${|\frac{d{\lambda }^{(n)}}{d\lambda }|}_{{\lambda }_{c}}\,\sim \,{N}^{1/\nu }$. Near the critical point, ${|\frac{d{\lambda }^{(n)}}{d\lambda }|}_{{\lambda }_{c}}\,\sim \,{|\frac{d{\xi }_{S}^{2}}{d\lambda }|}_{{\rm{\max }}}$, which implies that *θ* = 1/*ν*. So, the divergence exponent of ${|\frac{d{\xi }_{S}^{2}}{d\lambda }|}_{{\rm{\max }}}$ is related to the critical exponent of the correlation length.

Because we calculated the spin squeezing parameter at the GS, Eq. (17) does not contain parameter *J*. It is obvious that the spin squeezing parameter for the thermal state of a block, ${\rho }_{th}=\frac{1}{Z}\,\sum _{i=1}^{4}\,{e}^{-{E}_{i}/T}|{e}_{i}\rangle \langle {e}_{i}|$, depends on parameters *J*, *λ* and the temperature *T* as follows

18 $${\xi }_{S}^{2}(J,\lambda ,T)=1+\frac{-1+{{\rm{e}}}^{\frac{2J\sqrt{1+{\lambda }^{2}}}{T}}}{(1+{{\rm{e}}}^{\frac{2J\sqrt{1+{\lambda }^{2}}}{T}})\sqrt{1+{\lambda }^{2}}}.$$

**Figure 6:**
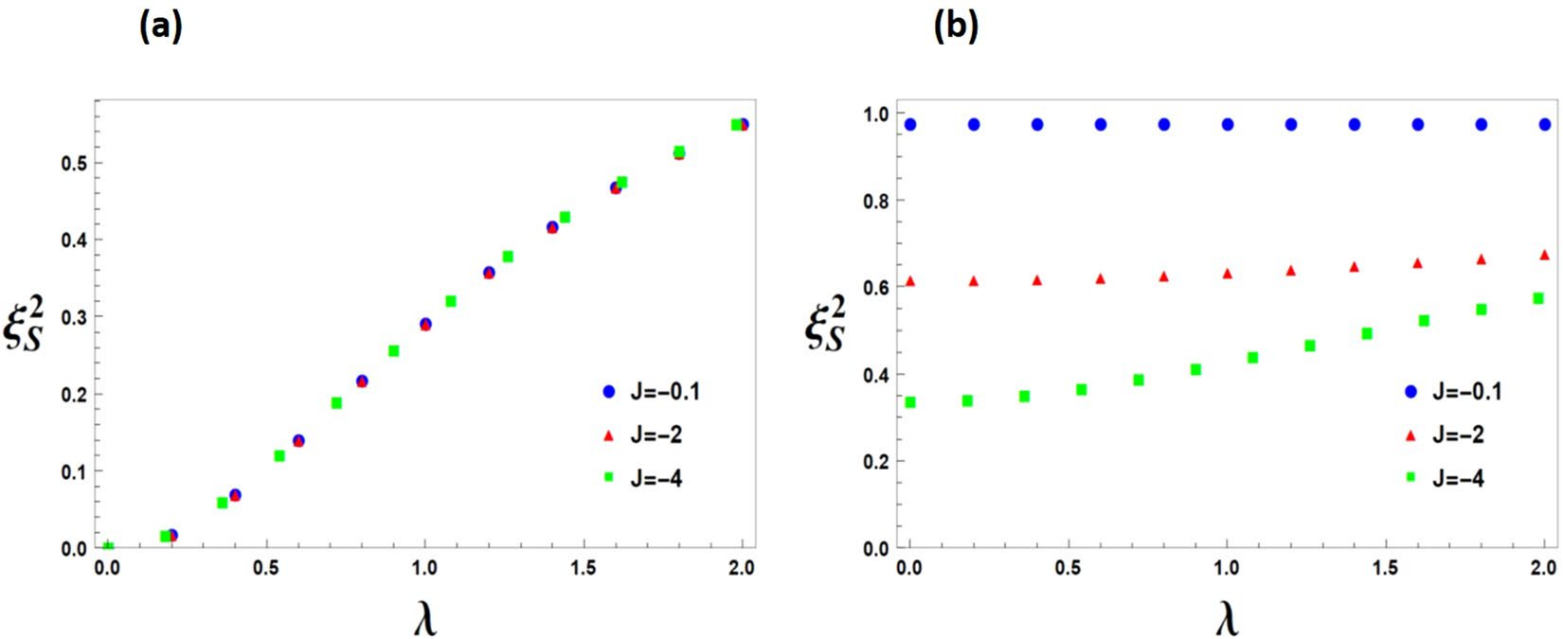
The spin squeezing parameter ${\xi }_{S}^{2}$ with different values of the parameter *J*, versus the transverse magnetic field *λ*, at (**a**) *T* = 0.0001 and (**b**) *T* = 5.

In Fig. 6, we have plotted thermal spin squeezing parameter, ${\xi }_{S}^{2}(J,\lambda ,T)$, for two cases of low and high temperatures. Clearly, at low temperatures, the spin squeezing parameter does not depend on parameter *J*, while it depends on *J* at high temperatures.

## Renormalization of the XXZ Model with DM Interaction

**Figure 7:**
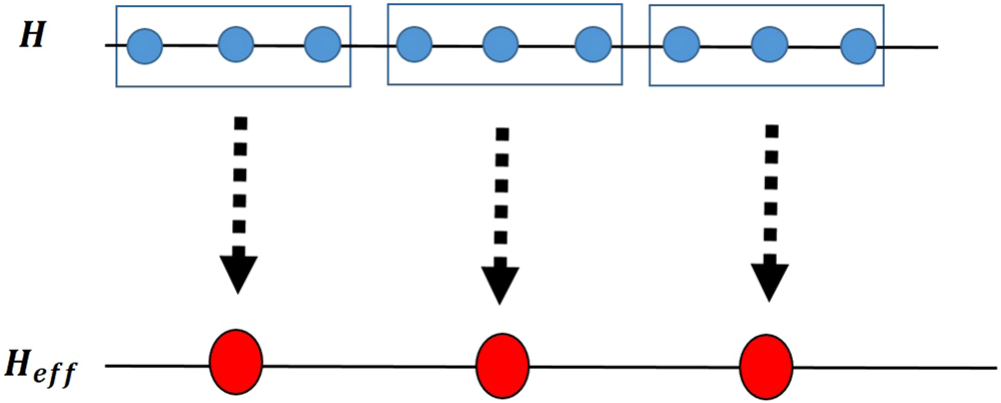
Representation of the Kadanoff’s block QRG method by decomposing a one-dimensional spin chain into three-site blocks.

The Hamiltonian of the one-dimensional anisotropic XXZ model with DM interaction in the *z*-direction on a periodic chain of *N* spin-1/2 is

19 $$H=\frac{J}{4}\,\sum _{i=1}^{N}\,[{\sigma }_{i}^{x}{\sigma }_{i+1}^{x}+{\sigma }_{i}^{y}{\sigma }_{i+1}^{y}+{\rm{\Delta }}{\sigma }_{i}^{z}{\sigma }_{i+1}^{z}+D({\sigma }_{i}^{x}{\sigma }_{i+1}^{y}-{\sigma }_{i}^{y}{\sigma }_{i+1}^{x})],$$

where *J* is the exchange coupling constant, Δ is the anisotropy parameter and *D* is the strength of the *z*-component of the DM interaction. To obtain a self-similar Hamiltonian after each QRG step, the chain is divided into three-site blocks, as shown in Fig. 7.

The inter-block Hamiltonian for the three site block and the corresponding eigenstates and eigenenergies are given in Appendix A of ref. ^70^. It has two degenerate ground states as

20 $$\begin{array}{rcl}|{\psi }_{0}\rangle  & = & \frac{1}{\sqrt{2q(q+{\rm{\Delta }}\mathrm{)(1}+{D}^{2})}}[\mathrm{(2(}{D}^{2}+\mathrm{1)}|\downarrow \,\downarrow \,\uparrow \rangle \\  &  & -\,\mathrm{(1}-iD)({\rm{\Delta }}+q)|\downarrow \,\uparrow \,\downarrow \rangle -2[2iD+({D}^{2}-\mathrm{1)}]|\uparrow \,\downarrow \,\downarrow \rangle ],\end{array}$$

21 $$\begin{array}{rcl}|{\psi ^{\prime} }_{0}\rangle  & = & \frac{1}{\sqrt{2q(q+{\rm{\Delta }}\mathrm{)(1}+{D}^{2})}}[\mathrm{2(}{D}^{2}+\mathrm{1)}|\downarrow \,\uparrow \,\uparrow \rangle \\  &  & -\,\mathrm{(1}-iD)({\rm{\Delta }}+q)|\uparrow \,\downarrow \,\uparrow \rangle -2[2iD+({D}^{2}-\mathrm{1)}]|\uparrow \,\uparrow \,\downarrow \rangle ],\end{array}$$

where $q=\sqrt{{{\rm{\Delta }}}^{2}+8(1+{D}^{2})}$. The first order correction of the effective Hamiltonian of the renormalized chain can be obtained as

22 $${H}_{eff}=\frac{J^{\prime} }{4}\,\sum _{i=1}^{N}\,[{\tilde{\sigma }}_{i}^{x}{\tilde{\sigma }}_{i+1}^{x}+{\tilde{\sigma }}_{i}^{y}{\tilde{\sigma }}_{i+1}^{y}+{\rm{\Delta }}^{\prime} {\tilde{\sigma }}_{i}^{z}{\tilde{\sigma }}_{i+1}^{z}+D^{\prime} ({\tilde{\sigma }}_{i}^{x}{\tilde{\sigma }}_{i+1}^{y}-{\tilde{\sigma }}_{i}^{y}{\tilde{\sigma }}_{i+1}^{x})],$$

where *J*′, Δ′ and *D*′ are the new scaled coupling constants given by the following recursion relations

23 $$J^{\prime} =J{(\frac{2}{q})}^{2}(1+{D}^{2}),\,D^{\prime} =D,\,{\rm{\Delta }}^{\prime} =\frac{{\rm{\Delta }}}{1+{D}^{2}}{(\frac{{\rm{\Delta }}+q}{4})}^{2}.$$

The Hamiltonian (22) is similar to the original Hamiltonian, i.e. Eq. (19). By considering ${\rm{\Delta }}^{\prime} ={\rm{\Delta }}\equiv {{\rm{\Delta }}}_{c}$ we can obtain stable and unstable fixed points of QRG. The critical fixed point is ${{\rm{\Delta }}}_{c}=\sqrt{1+{D}^{2}}$. The model represents the spin fluid phase for ${\rm{\Delta }} < \sqrt{1+{D}^{2}}$ and the Neel phase for ${\rm{\Delta }} > \sqrt{1+{D}^{2}}$.

## Renormalization of the Spin Squeezing in the XXZ Model with DM Interaction

In this subsection, we calculate the spin squeezing parameter of the XXZ model with DM interaction by considering one of the degenerated GSs. For |*ψ*_0_〉, one obtains

24 $${\xi }_{S}^{2}=\tfrac{3{\rm{\Delta }}\sqrt{8{D}^{2}+{{\rm{\Delta }}}^{2}+8}-8\sqrt{8{D}^{2}+{{\rm{\Delta }}}^{2}+8}+16{D}^{2}+3{{\rm{\Delta }}}^{2}-8{\rm{\Delta }}+32}{3({\rm{\Delta }}\sqrt{8{D}^{2}+{{\rm{\Delta }}}^{2}+8}+8{D}^{2}+{{\rm{\Delta }}}^{2}+8)},$$

The results are the same if one uses $|{\psi ^{\prime} }_{0}\rangle$.

**Figure 8:**
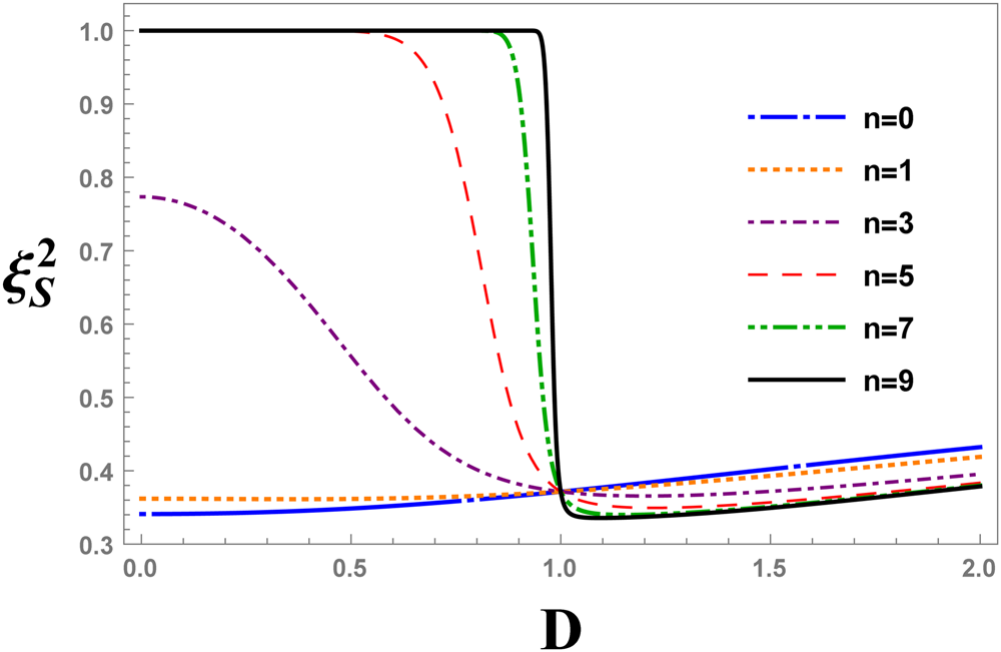
The evolution of the spin squeezing parameter after the QRG iterations *n*, versus the DM interaction *D*, at ${\rm{\Delta }}=\sqrt{2}$ in the XXZ model with DM interaction.

**Figure 9:**
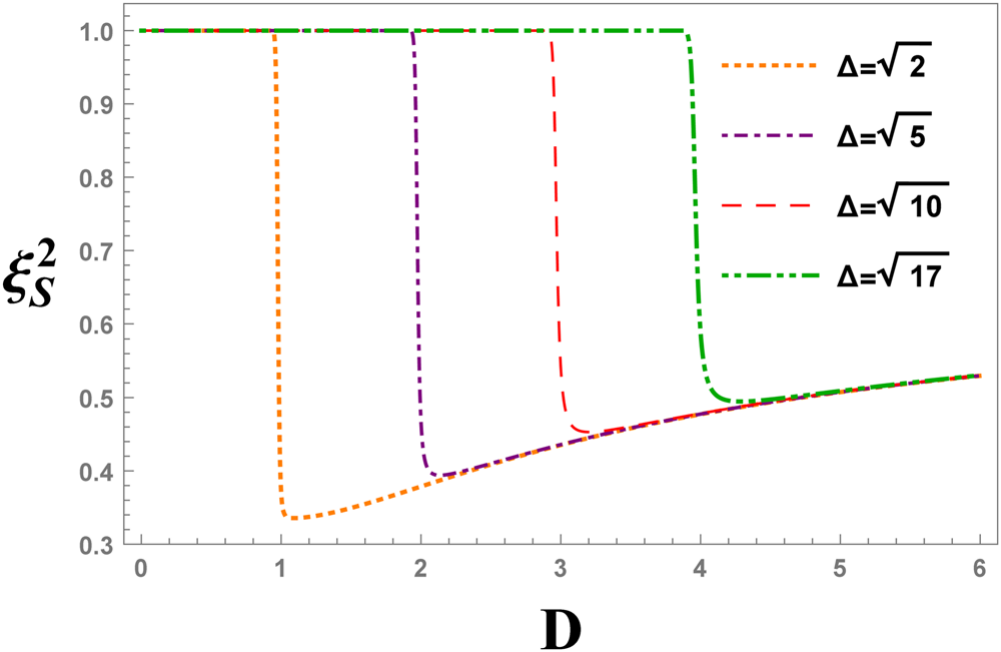
The evolution of the spin squeezing parameter, ${\xi }_{S}^{2}$, after different values of the anisotropy parameter, versus the DM interaction *D*, at ninth QRG step in the XXZ model with DM interaction.

In Fig. 8, the evolution of the spin squeezing parameter, ${\xi }_{S}^{2}$, with QRG steps is plotted as a function of the strength of the DM interaction at ${\rm{\Delta }}=\sqrt{2}$. All plots cross at *D* = 1, that is in correspondence with the fixed point of the recursion relation ${{\rm{\Delta }}}_{c}=\sqrt{1+{D}^{2}}$. The scale-free point of Fig. 8, gives the QCP. By increasing the scale of the system, i.e. some QRG iterations, ${\xi }_{S}^{2}$ drops suddenly from the value 1 for *D* < 1 to the value 0 for *D* > 1. In Fig. 9, the spin squeezing parameter at ninth QRG step is plotted as a function of *D* for different values of the anisotropy parameter, ${\rm{\Delta }}=\sqrt{2},\sqrt{5},\sqrt{10},\sqrt{17}$. The sudden change of graphs at points *D* = 1, 2, 3, 4 corresponding to their anisotropy parameters ${\rm{\Delta }}=\sqrt{2},\sqrt{5},\sqrt{10},\sqrt{17}$ confirms the power of spin squeezing measure in detecting QCPs.

**Figure 10:**
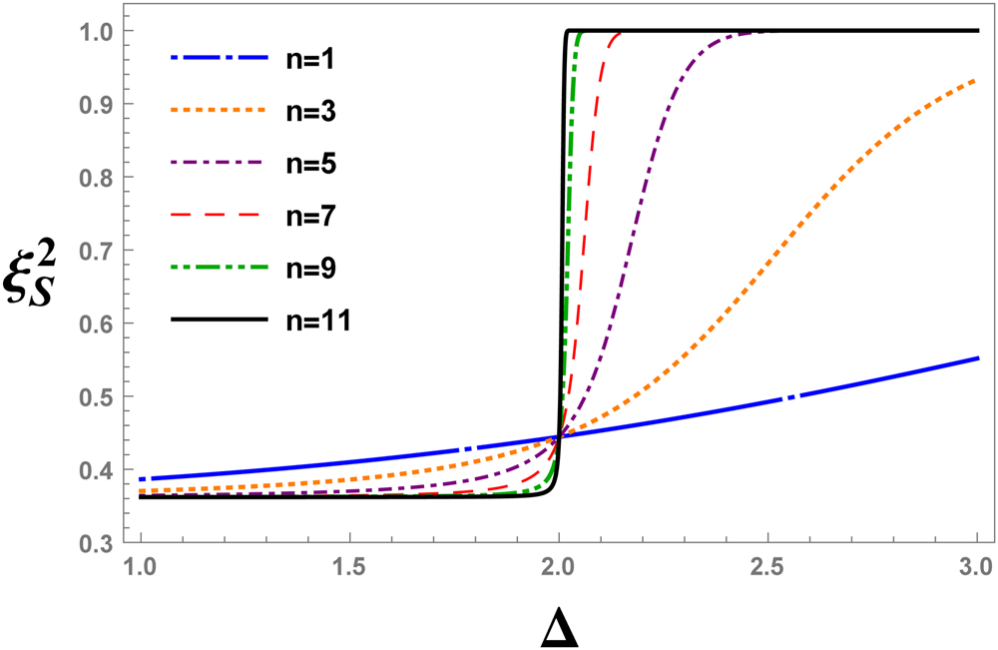
The evolution of the spin squeezing parameter after QRG iteration *n*, versus the anisotropy parameter Δ, at $D=\sqrt{3}$ in the XXZ model with DM interaction.

**Figure 11:**
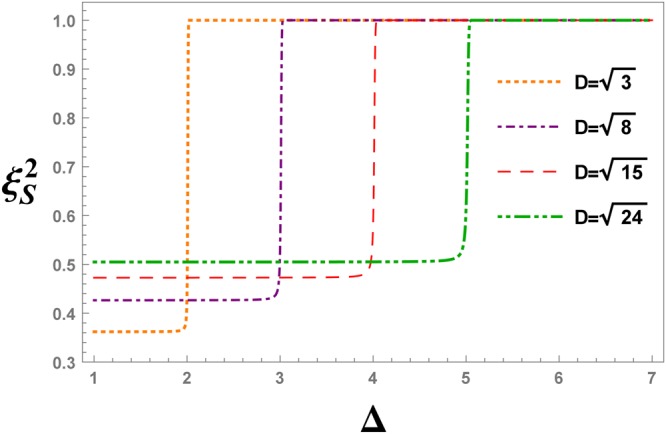
The evolution of the spin squeezing parameter ${\xi }_{S}^{2}$ under different values of the DM interaction, versus the anisotropy parameter Δ, at 11-th QRG step in the XXZ model with DM interaction.

Figures 10 and 11 have similar arguments except for that ${\xi }_{S}^{2}$ is plotted versus the anisotropy parameter, Δ. The fixed point of the curves in Fig. 10, i.e. Δ = 2, is consistent with the fixed value of the DM interaction, $D=\sqrt{3}$, and ${{\rm{\Delta }}}_{c}=\sqrt{1+{D}^{2}}$.

**Figure 12:**
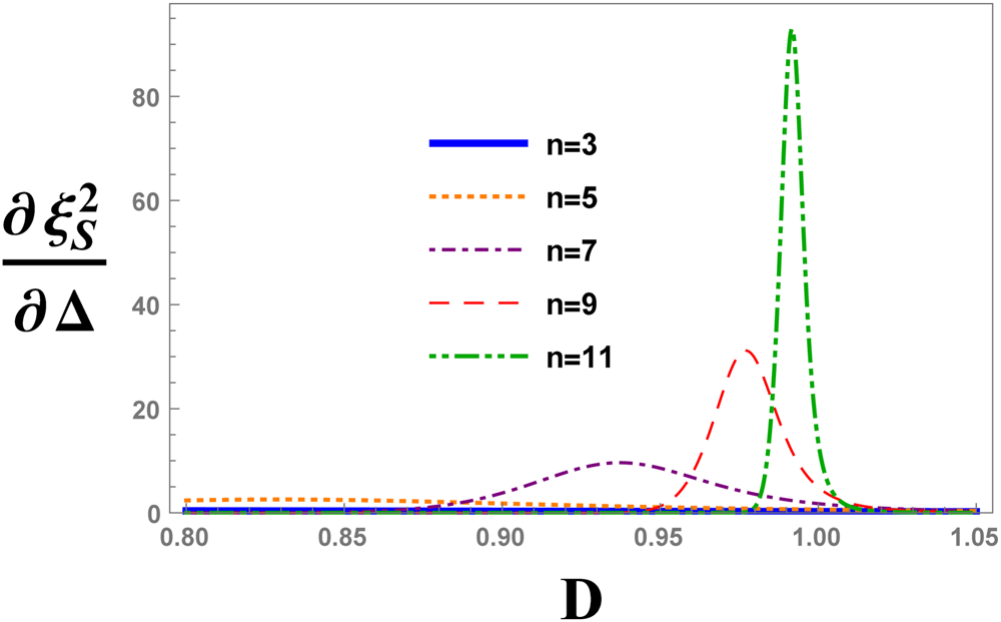
The QRG evolution of the first derivative of the spin squeezing parameter, ${\xi }_{S}^{2}$, with respect to the anisotropy parameter, Δ, versus the DM interaction *D* in the XXZ model with DM interaction, evaluated at ${\rm{\Delta }}=\sqrt{2}$.

For more details of the critical behaviour, we plot the evolution of the first derivative of ${\xi }_{S}^{2}$ with respect to the strength of the DM interaction, i.e. $\frac{d{\xi }_{S}^{2}}{d{\rm{\Delta }}}$, at ${\rm{\Delta }}=\sqrt{2}$ in Fig. 12 for some QRG steps. It can be seen from Fig. 12, there is a peak for each QRG step with position *D*_max_ that tends to *D* = 1 at the thermodynamic limit.

**Figure 13:**
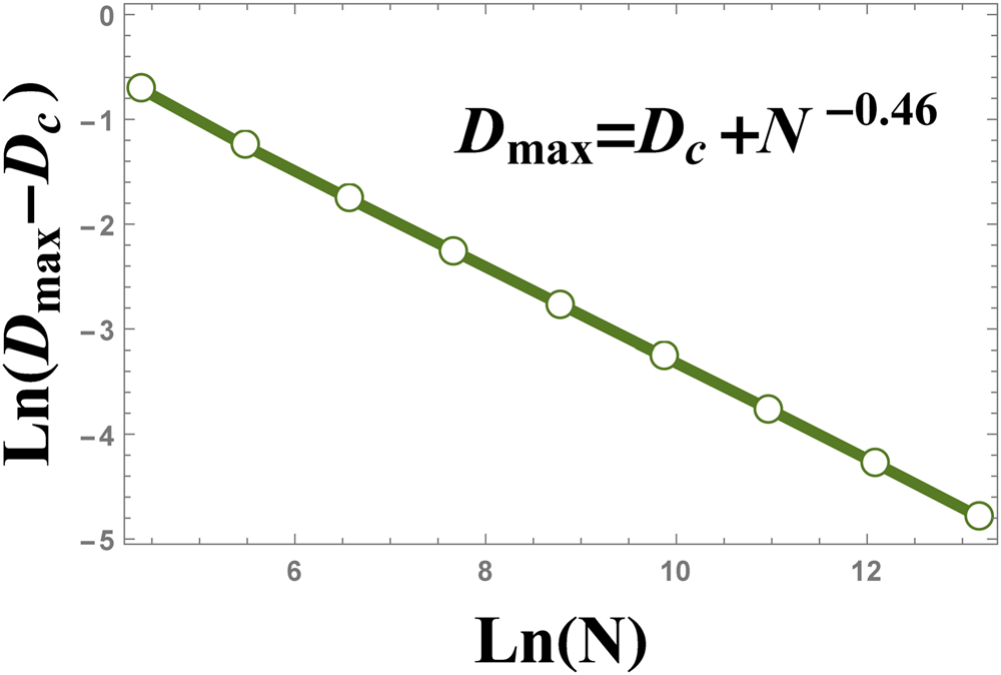
The scaling behaviour of $|{D}_{{\rm{\max }}}-{D}_{c}|$ versus the size of the system *N*, where *D*_max_ is the position of the peak in Fig. 12.

In Fig. 13, the absolute value of *D*_max_ − *D*_*c*_ is plotted versus the size of the system. Figure 13 illustrates that the position of the maximum of $\frac{d{\xi }_{S}^{2}}{d{\rm{\Delta }}}$, i.e. *D*_max_, scales as $|{D}_{{\rm{\max }}}-{D}_{c}|={N}^{-\theta }$, where *θ* = −0.46.

**Figure 14:**
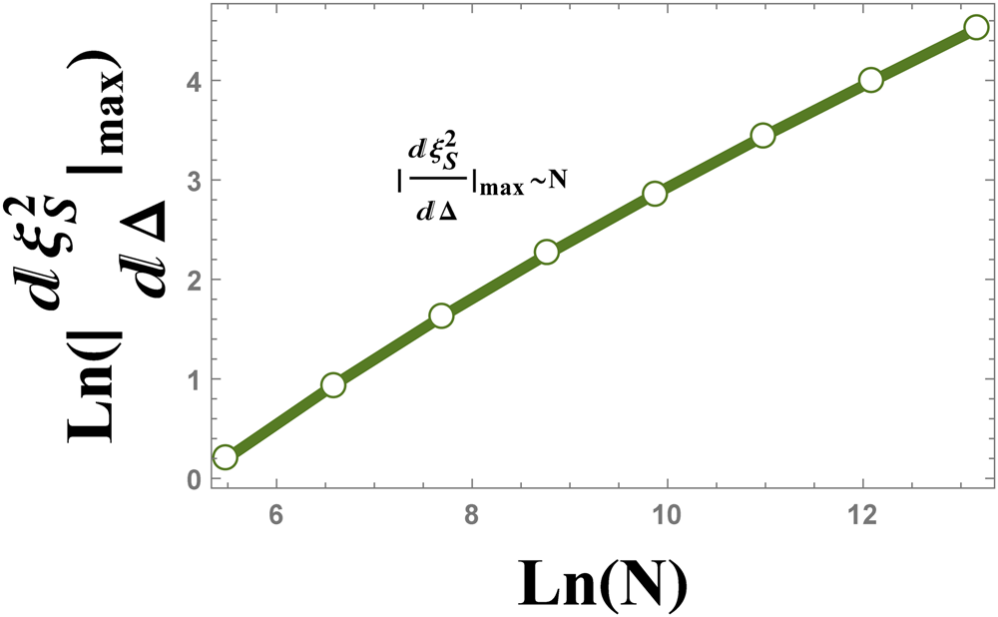
The logarithmic plot of the absolute value of ${\tfrac{d{\xi }_{S}^{2}}{d{\rm{\Delta }}}|}_{{\rm{\max }}}$ versus the size of the system, *N*. The plot shows the scaling behaviour ${|\tfrac{d{\xi }_{S}^{2}}{d{\rm{\Delta }}}|}_{{\rm{\max }}}\,\sim \,{N}^{0.56}$.

The numerical results plotted in Fig. 14, show the behaviour of the absolute value of the maximum of $\frac{d{\xi }_{S}^{2}}{d{\rm{\Delta }}}$ versus the size of the system *N*, as ${|\frac{d{\xi }_{S}^{2}}{d{\rm{\Delta }}}|}_{{D}_{{\rm{\max }}}}\,\sim \,{N}^{0.56}$.

**Figure 15:**
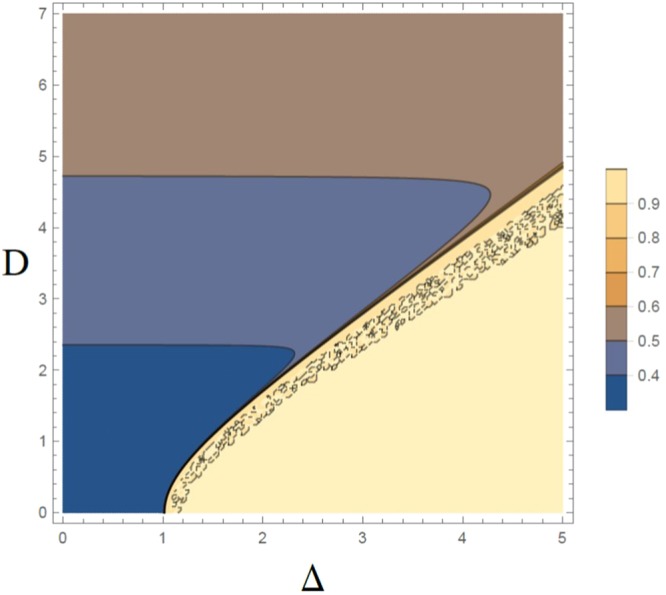
The evolution of the spin squeezing parameter, ${\xi }_{S}^{2}$, in the parameter space (Δ, *D*), for ninth QRG step in the XXZ model with DM interaction.

In Fig. 15, ${\xi }_{S}^{2}$ has been ploted in the parameter space (Δ, *D*), for the ninth QRG step. From the graph, the critical line of this model, i.e. ${{\rm{\Delta }}}_{c}=\sqrt{1+{D}^{2}}$, can be observed, clearly.

## Renormalization of the XXZ Model

The Hamiltonian of the one dimensional anisotropic XXZ model on a periodic *N* spin-1/2 chain is

25 $$H=\frac{J}{4}\,\sum _{i=1}^{N}\,[{\sigma }_{i}^{x}{\sigma }_{i+1}^{x}+{\sigma }_{i}^{y}{\sigma }_{i+1}^{y}+{\rm{\Delta }}{\sigma }_{i}^{z}{\sigma }_{i+1}^{z}].$$

The results of the QRG calculation on this model can be extracted by substituting *D* = 0 into the equations of the previous model in Sect. The self-similar effective Hamiltonian after one QRG step is

26 $${H}_{eff}=\frac{J^{\prime} }{4}\,\sum _{i=1}^{N}\,[{\tilde{\sigma }}_{i}^{x}{\tilde{\sigma }}_{i+1}^{x}+{\tilde{\sigma }}_{i}^{y}{\tilde{\sigma }}_{i+1}^{y}+{\rm{\Delta }}^{\prime} {\tilde{\sigma }}_{i}^{z}{\tilde{\sigma }}_{i+1}^{z}],$$

The recursion relations of the QRG flow are

27 $$J^{\prime} =\frac{4J}{{{\rm{\Delta }}}^{2}+8},\,{\rm{\Delta }}^{\prime} ={\rm{\Delta }}{(\frac{{\rm{\Delta }}+\sqrt{{{\rm{\Delta }}}^{2}+8}}{4})}^{2},$$

where *J*′ and Δ′ are the renormalized couplings. By solving ${\rm{\Delta }}^{\prime} ={\rm{\Delta }}\equiv {{\rm{\Delta }}}_{c}$, the stable and unstable fixed points of the QRG equations can be obtained. The critical point of this model is located at Δ_*c*_ = 1

## Renormalization of the Spin Squeezing in the XXZ Model

We can calculate the spin squeezing parameter of the XXZ model for the GS by substituting *D* = 0 into Eq. (24). Then, one obtains for ${\xi }_{S}^{2}$

28 $${\xi }_{S}^{2}=\frac{3{{\rm{\Delta }}}^{2}+3\sqrt{{{\rm{\Delta }}}^{2}+8}\,{\rm{\Delta }}-8\sqrt{{{\rm{\Delta }}}^{2}+8}-8{\rm{\Delta }}+32}{3({{\rm{\Delta }}}^{2}+\sqrt{{{\rm{\Delta }}}^{2}+8}\,{\rm{\Delta }}+8)},$$

**Figure 16:**
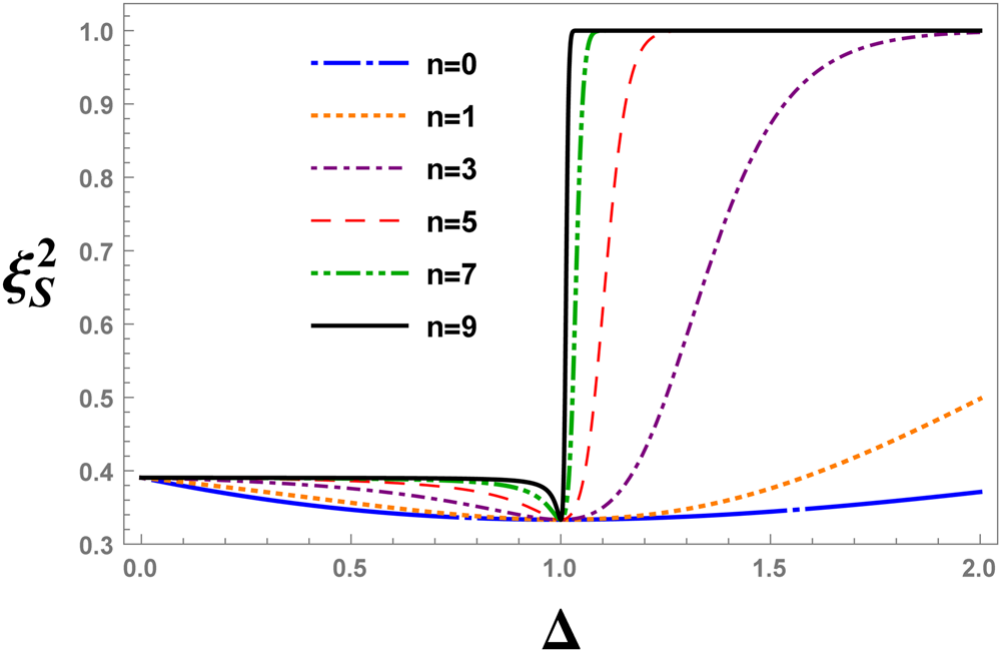
The evolution of the spin squeezing parameter, ${\xi }_{S}^{2}$, after QRG iteration *n*, versus the anisotropy parameter Δ in the XXZ model.

In Fig. 16, the evolution of the spin squeezing parameter after *n*-th QRG steps is plotted as a function of the anisotropy parameter, Δ. All plots cross at Δ = 1, that is in correspondence with a previous study^71^. The scale-free point of Fig. 16, gives the QCP. By increasing the scale of the system, i.e. after QRG iterations, the spin squeezing parameter changes suddenly from a value between zero and one for Δ < 1 to one for Δ > 1.

**Figure 17:**
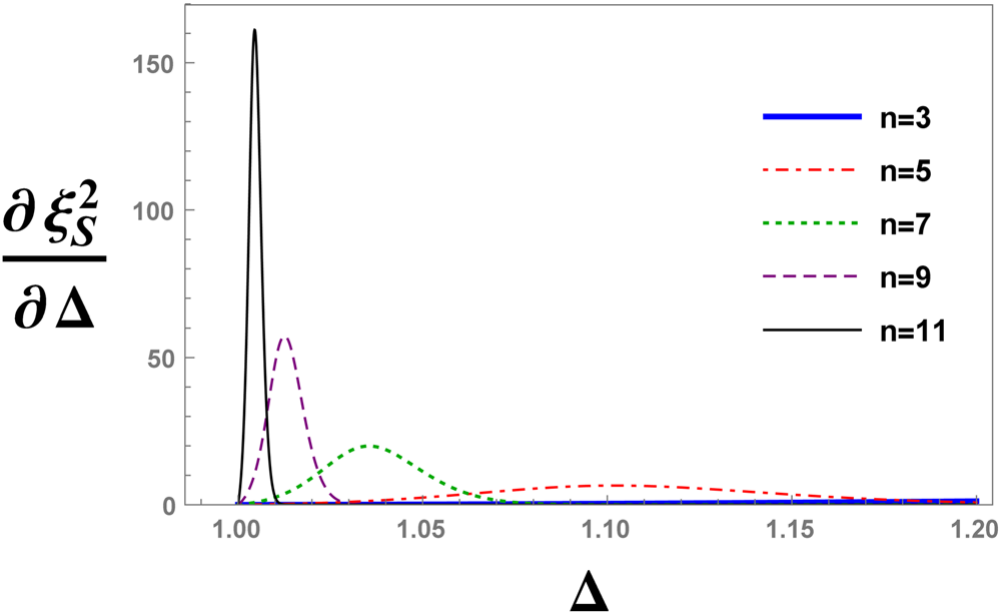
The evolution of the first derivative of the spin squeezing parameter, ${\xi }_{S}^{2}$, with respect to the anisotropy parameter Δ, under QRG iterations *n* in the XXZ model.

For more details of the critical behaviour, we study the evolution of the first derivative of the spin squeezing parameter with QRG steps as a function of the anisotropy parameter, i.e.; $\frac{d{\xi }_{S}^{2}}{d{\rm{\Delta }}}$, as is plotted in Fig. 17. As it can be seen from the figure, there is a peak for each QRG step with position Δ_max_ that tends to Δ = 1 at the thermodynamic limit.

**Figure 18:**
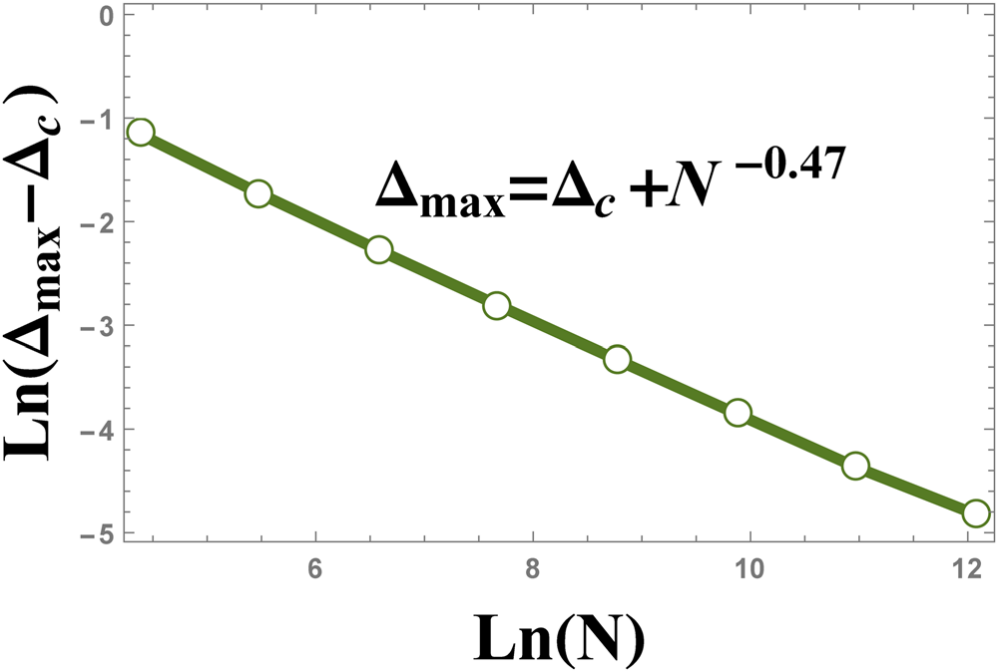
The scaling behaviour of $|{{\rm{\Delta }}}_{{\rm{\max }}}-{{\rm{\Delta }}}_{c}|$ versus the size of the system *N*, where Δ_max_ is the position of the peak in Fig. 16.

In Fig. 18, the absolute value of Δ_max_ − Δ_*c*_ is plotted versus the size of the system. Figure 18 illustrates that Δ_max_ scales as $|{{\rm{\Delta }}}_{{\rm{\max }}}-{{\rm{\Delta }}}_{c}|={N}^{-\theta }$, where *θ* = −0.47.

**Figure 19:**
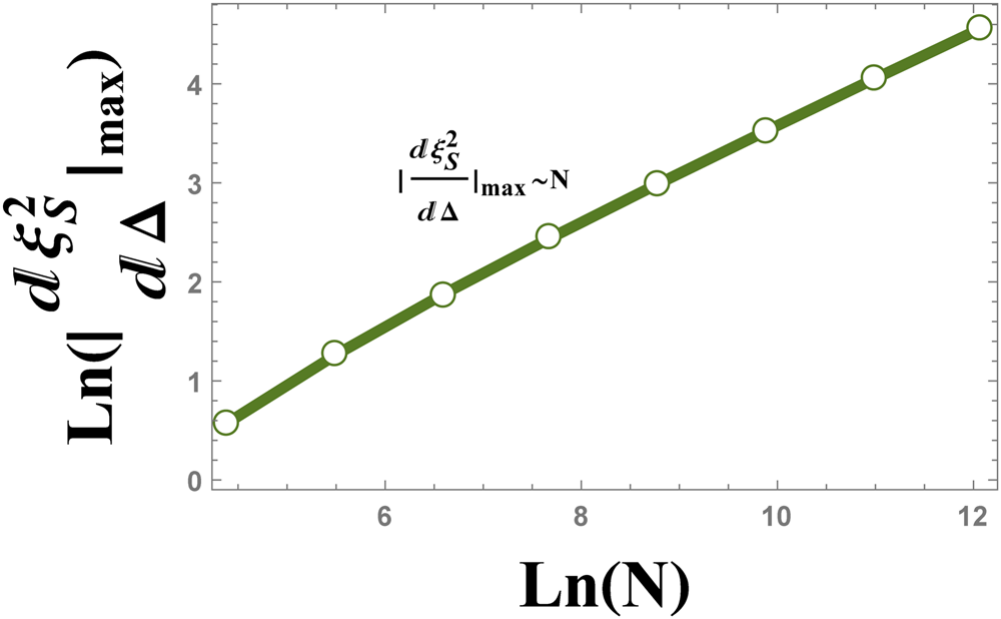
The logarithmic plot of the absolute value of ${\tfrac{d{\xi }_{S}^{2}}{d{\rm{\Delta }}}|}_{{\rm{\max }}}$ versus the size of the system, *N*. The plot shows the scaling behaviour ${|\tfrac{d{\xi }_{S}^{2}}{d{\rm{\Delta }}}|}_{{\rm{\max }}}\,\sim \,{N}^{0.51}$.

The numerical results that is plotted in Fig. 19, show the behaviour of the absolute value of the maximum of $\frac{d{\xi }_{S}^{2}}{d{\rm{\Delta }}}$ versus the size of the system *N*, as ${|\frac{d{\xi }_{S}^{2}}{d{\rm{\Delta }}}|}_{{{\rm{\Delta }}}_{{\rm{\max }}}}\,\sim \,{N}^{0.51}$.

The simple XXZ model has a critical line that can not be detected without imposing the effect of boundary conditions on each block Hamiltonian. When we add the DM interaction to the XXZ model, its critical behaviors become detectable by studying the spin squeezing parameter, which are in agreement with the other analytical or numerical results.

The XXZ model on a periodic chain of N spin-1/2, has a critical line for $0\leqslant {\rm{\Delta }}\leqslant 1$, which is not detected by our approach. Here, we use another QRG method which uses the concept of quantum groups, proposed by Delgado, *et al*.^68^. The renormalization of entanglement in this method used by Kargarian *et al*.^24^, leads to the detection of the critical line of the XXZ model. This approach is suggested by the fact that quantum groups describe symmetries in the presence of appropriate boundary conditions, e.g. boundary magnetic fields. The main idea of this method, is to add the boundary magnetic fields to the Hamiltonian, that cancel each other when considering all blocks. The open spin chain Hamiltonian is defined as:

29 $$H=\tfrac{J}{4}\,\sum _{i=1}^{N}\,[{\sigma }_{i}^{x}{\sigma }_{i+1}^{x}+{\sigma }_{i}^{y}{\sigma }_{i+1}^{y}+(\tfrac{q+{q}^{-1}}{2}){\sigma }_{i}^{z}{\sigma }_{i+1}^{z}-(\tfrac{q-{q}^{-1}}{2})({\sigma }_{i}^{z}-{\sigma }_{i+1}^{z})],$$

where *q* is an arbitrary complex parameter. To get a self-similar Hamiltonian, the system is divided to three spin blocks as shown in Fig. 8. The inter-block Hamiltonian and the intrablock Hamiltonian are as follows:

30 $$\begin{array}{rcl}{h}_{I}^{B} & = & \tfrac{J}{4}\,\sum _{i=1}^{N}\,[\begin{array}{l}{\sigma }_{1,I}^{x}{\sigma }_{2,I}^{x}+{\sigma }_{2,I}^{x}{\sigma }_{3,I}^{x}+{\sigma }_{1,I}^{y}{\sigma }_{2,I}^{y}+{\sigma }_{2,I}^{y}{\sigma }_{3,I}^{y}\\ +\,(\tfrac{q+{q}^{-1}}{2})({\sigma }_{1,I}^{z}{\sigma }_{2,I}^{z}+{\sigma }_{2,I}^{z}{\sigma }_{3,I}^{z})-(\tfrac{q-{q}^{-1}}{2})({\sigma }_{1,I}^{z}-{\sigma }_{3,I}^{z})\end{array}]\\ {H}^{BB} & = & \tfrac{J}{4}\,\sum _{I=1}^{N/3}\,[\begin{array}{l}{\sigma }_{3,I}^{x}{\sigma }_{1,I+1}^{x}+{\sigma }_{3,I}^{y}{\sigma }_{1,I+1}^{y}+(\tfrac{q+{q}^{-1}}{2}){\sigma }_{3,I}^{z}{\sigma }_{1,I+1}^{z}\\ -\,(\tfrac{q-{q}^{-1}}{2})({\sigma }_{3,I}^{z}-{\sigma }_{1,I+1}^{z})\end{array}]\end{array}$$

The two fold degenerated GSs of the block Hamiltonian are

31 $$\begin{array}{rcl}|{\psi }_{0}\rangle  & = & \tfrac{1}{\sqrt{2(q+{q}^{-1}+1)}}[\,-{q}^{1/2}|\uparrow \,\uparrow \,\downarrow \rangle +({q}^{1/2}+{q}^{-1/2})|\uparrow \,\downarrow \,\uparrow \rangle -{q}^{-1/2}|\downarrow \,\uparrow \,\uparrow \rangle ],\\ |{\psi ^{\prime} }_{0}\rangle  & = & \tfrac{1}{2(q+{q}^{-1}+1)}[\,-{q}^{1/2}|\uparrow \,\downarrow \,\downarrow \rangle +({q}^{1/2}+{q}^{-1/2})|\downarrow \,\uparrow \,\downarrow \rangle -{q}^{-1/2}|\downarrow \,\downarrow \,\uparrow \rangle ].\end{array}$$

The effective Hamiltonian with the renormalized coupling constants is

32 $${H}_{eff}=\tfrac{J^{\prime} }{4}\,\sum _{i}^{N/3}\,[{\sigma }_{i}^{x}{\sigma }_{i+1}^{x}+{\sigma }_{i}^{y}{\sigma }_{i+1}^{y}+(\tfrac{q^{\prime} +{q^{\prime} }^{-1}}{2}){\sigma }_{i}^{z}{\sigma }_{i+1}^{z}-(\tfrac{q^{\prime} -{q^{\prime} }^{-1}}{2})({\sigma }_{i}^{z}-{\sigma }_{i+1}^{z})],$$

where

33 $$q^{\prime} =q,\,J^{\prime} ={(\frac{q+{q}^{-1}+2}{2(q+{q}^{-1}+1)})}^{2}J.$$

Due to these recursion relations, the coupling constant *q* does not evolve under QRG method. Assuming ${\rm{\Delta }}=\frac{q+{q}^{-1}}{2}$, for *q* being a complex number and 0 < Δ < 1), the coupling constant *q* can be written as a pure phase. We can calculate the spin squeezing parameter of the GS, |*ψ*_0_〉. The result is ${\xi }_{S}^{2}=\frac{1}{3}$. Because of the fixed value of the spin squeezing parameter in the region 0 < Δ < 1, it does not evolve under QRG transformation which is a signature of the critical line of the XXZ model in this region.

It is also noticable that the size scaling of the $\frac{d{\xi }_{S}^{2}}{d{\rm{\Delta }}}$ in Figs 13, 14, 18 and 19 is not logarithmic as may be expected based on the fact that the corresponding QPT is Kosterlitz-Thouless type. The power scaling behaviour was also seen in the maximum of the fidelity susceptibility, which is in close correspondence with $\frac{d{\xi }_{S}^{2}}{d{\rm{\Delta }}}$ in our study, while the maximum in the entanglement entropy itself shows logarithmic scaling^72^. This suggests that the scaling behaviour is measure and method dependent.

The main requirement of the QRG method is that after renormalization the effective Hamiltonian must be similar to the original Hamiltonian, i.e. the system is renormalizable. It is not clear that if renormalization can yield the physical properties of QPTs precisely for every model system, thought some models are not even renormalizable. The models we have selected here are particular ones where the method works confidently. Counter example are spin-1 XYZ Heisenberg model with DM interaction or spin-1 isotropic bilinear biquadratic Heisenberg model in the presence of magnetic field. For summary, we have gathered some examples of renormalizable spin models and QPT indicators in QRG approach in Table 1. Investigation of critical behaviours of a non trivial example such as XXZ Heisenberg model with single ion anisotropy in a one dimensional spin 1 chain, is under debate by authors of this paper.

**Table 1:** Various spin models and their relative QPT indicators in QRG approach.

Model	Indicator	References
ITF	Concurrence	^23^
XXZ	Concurrence	^24^
XYZ in a transverse field	Structure factor	^27^
XXZ with DM interaction	Trance distance discord	^29^
ITF	Geometric quantum discord	^30^
ITF	Geometric phase	^31^
XXZ with single ion anisotropy (spin 1)	Fidelity	^32^
XY	Monogamy relations of Negativity	^33^
ITF, XXZ, XXZ with DM interaction	Spin squeezing parameter	current study

## Conclusion

In this work the relation between the spin squeezing and QPT is addressed based on the QRG procedure. We have used the idea of QRG to study the quantum information properties of the ITF, the XXZ with and without DM interaction. Spin squeezing is used as the witness of quantum entanglement. In order to explore the critical behaviour of the spin models, the evolution of the spin squeezing parameter with renormalization of the model is examined. As the number of QRG iterations increases, the spin squeezing parameter develops its saturated values in both sides of the QCP. The first derivative of the spin squeezing parameter diverges close to the QCP as the scale of the system becomes larger. In the near vicinity of the QCP, the critical exponent of the spin squeezing parameter is in correspondence with the critical exponent of the correlation length.
